# Is It Antiphospholipid Syndrome?

**DOI:** 10.1155/2010/932157

**Published:** 2011-01-13

**Authors:** Maria Chiara Ditto, Marco Antivalle, Matteo Badini, Michele Battellino, Chiara Cogliati, Piercarlo Sarzi-Puttini

**Affiliations:** ^1^Rheumatology Unit, L. Sacco University Hospital, Via G.B. Grassi 74, 20157 Milano, Italy; ^2^Internal Medicine Unit, L. Sacco University Hospital, Via G.B. Grassi 74, 20157 Milano, Italy

## Abstract

The diagnosis of bacterial endocarditis remains a challenge, as nearly half of cases develop in the absence of preexistent heart disease and known risk factors. Not infrequently, a blunted clinical course at onset can lead to erroneous diagnoses. We present the case of a 47-year-old previously healthy man in which a presumptive diagnosis of antiphospholipid syndrome was made based on the absence of echocardiographically detected heart involvement, a negative blood culture, normal C-reactive protein (CRP) levels, a positive lupus anticoagulant (LAC) test, and evidence of splenic infarcts. The patient eventually developed massive aortic endocarditic involvement, with blood cultures positive for *Streptococcus bovis*, and was referred for valvular replacement. This case not only reminds us of the diagnostic challenges of bacterial endocarditis, but also underlines the need for a critical application of antiphospholipid syndrome diagnostic criteria.

## 1. Introduction

Antiphospholipid syndrome (APS) and infective endocarditis (IE) are difficult-to-diagnose diseases and share several clinical features. To increase the probability of excluding temporary infection-associated antibodies, current diagnostic criteria for APS require Antiphospholipid Antibodies (APA) positivity on two separate occasions at least twelve weeks apart [1]. We describe here the case of a 47-year-old male in whom an erroneous diagnosis of presumptive APS, waiting for confirmatory laboratory test, was made, leading to a potentially dangerous delay in the treatment of infective endocarditis. The diagnostic challenges presented by this patient are discussed.

## 2. Case Report

A 47-year-old previously healthy man was admitted to our department because of arthralgias, remittent fever, and pericardial effusion. His medical history was unremarkable. Approximately one year earlier he had had an episode of paroxysmal atrial fibrillation with pharmacological restoration of sinus rhythm. For the past six months before admission, he reported remittent low-grade fever, tachycardia, night sweats, cervical pain, and migratory arthralgias with transient joint swelling. The patient was evaluated by an internal medicine specialist. Blood test revealed WBC 7.000/mmc, Hb 14.0 g/dL, and C-reactive protein (CRP) 9.3 mg/L. C3 and C4 complement components were within normal limits, and antinuclear antibodies were absent. A small IgG-K monoclonal gammopathy (7.94 g/L) was detected, with negative Bence-Jones proteinuria. A blood culture was negative. Silica clotting time, lupus anticoagulant (LAC) screening (LAR 1.97), and confirmatory tests (LAR 1.32) were positive. No murmur was found on cardiac examination. An electrocardiogram showed normal sinus rhythm, and at transthoracic echocardiogram (TTE), only trivial tricuspidal and aortic regurgitation was found. Routine chest X-ray was unremarkable, but a chest and abdomen CT scan showed tiny bilateral pleural effusions and an enlarged spleen with small parenchymal infarctions.

At rheumatological consultation, a confirmatory LAC test was positive. Anticardiolipin (aCL) and anti-*β*2 glycoprotein-I antibodies were absent. Screening tests for associated thrombophilic risk factors revealed the presence of MTHFR 677/CT mutation with hyperhomocysteinemia (19.1 *μ*mol/L). A preliminary diagnosis of APS, waiting for confirmatory LAC test over 12 weeks [1], was made, and treatment with acetylsalicylic acid 100 mg/day and etoricoxib 90 mg/day as needed, along with folic acid and vitamin B6 supplementation, was instituted, resulting in remission of arthralgias and amelioration of general well-being. Remitting fever sometimes with shivering persisted throughout the subsequent weeks. Three weeks later, he was admitted to an emergency department complaining of tachycardia and dyspnea. Body temperature was 38.5°C, heart rate was 105 bpm, and respiratory rate was 28/min. Blood pressure was 110/65 mmHg, and oxygen saturation was 96% while the patient was breathing ambient air; the remainder of the examination was normal. Inflammatory markers were slightly elevated. An echocardiogram was performed, showing only moderate pericardial effusion. Pleural fluid analysis showed absence of neoplastic cells and a leucocyte count lower than 250/mmc, and cultures were negative. Laboratory data showed a positive c-ANCA test (30 U/mL). IgM aCL antibodies were positive (64 MPL). LAC was not retested. The clinical picture was interpreted as an autoimmune pleuropericarditis, and treatment with aspirin 3 g daily and methylprednisolone (1 mg/kg/day) was instituted. 

**Figure 1:**
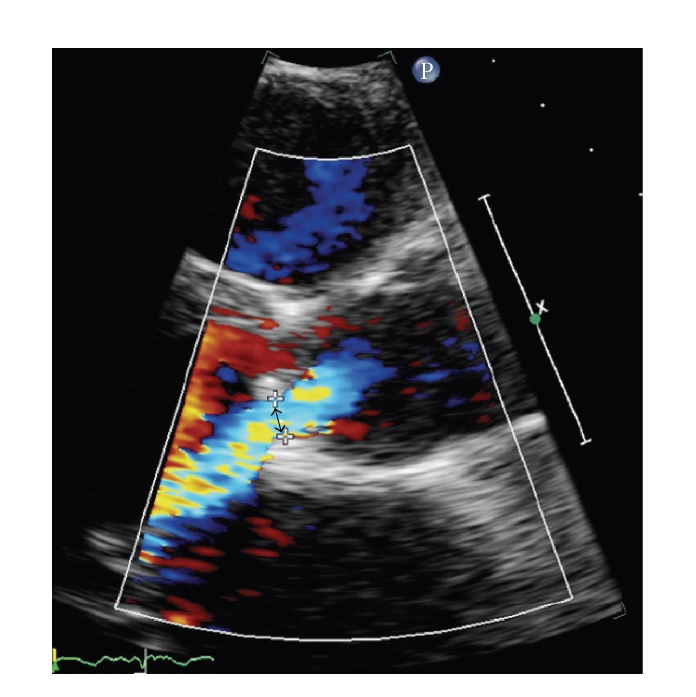
Color doppler evaluation of the aortic valve regurgitation: the arrow points to “vena contracta” of a central severe regurgitant jet.

**Figure 2:**
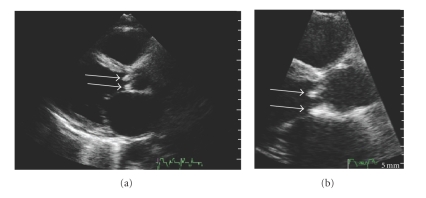
Transthoracic echocardiographic examination: parasternal long axis view. The arrows indicate two vegetations on the ventricular side of the aortic valve cusps (right and noncoronary).

At the time of admission to our department, 20 days later, the patient had remittent fever with daily spikes over 38.5°C. The pulse rate was 140 bpm, blood pressure was 120/85 mmHg, and respiratory rate was 24/min. Oxygen saturation was 94% while the patient was breathing ambient air. Acute-phase reactants were significantly increased (CRP = 83.3 mg/L). Blood and urine chemistries were otherwise within normal ranges. aCL and ANCA were absent. On physical examination, the patient appeared asthenic but not acutely ill. There was no cervical lymphadenopathy. The lower liver edge was palpated 2 to 3 cm below the right costal margin. Auscultation of the chest revealed bibasilar rales. There was a grade 3/6 systolic murmur and a grade 2/6 protodiastolic murmur. An electrocardiogram showed normal sinus rhythm with only nonspecific repolarization abnormalities. A transthoracic echocardiogram revealed a moderate-to-severe jet of aortic regurgitation (Figure 1), and three floating structures adherent to the aortic cusps, of which the greatest was 30 × 10 mm, were visualized (Figure 2). Moderate to severe mitral insufficiency due to annular dilation without significant mitral valve leaflets involvement was present, and moderate tricuspidal insufficiency with pulmonary hypertension (pulmonary artery pressure: 60 mmHg) was observed. Transesophageal echocardiogram confirmed the findings of the transthoracic examen, showing a large vegetation originating from the ventricular side of semilunar valves and protruding into the left ventricle during diastole. The patient was eventually referred to the heart surgery division. Intraoperatively, endocarditis affecting the ventricular side of the three aortic cusps and without annular involvement was confirmed. Aortic valve replacement and mitral valve ring annuloplasty were performed. Pleuritic (1800 ml) and pericardial effusions were removed. Blood cultures were positive for *Streptococcus bovis*, and the patient was treated with Gentamicin 240 mg/day and Ampicillin 12 gr/day, and long-term anticoagulation with warfarin was started. Finally, a colonoscopy was performed, as the association between *S*. *bovis* bacteremia or endocarditis and colonic neoplasia has been appreciated for many years, and a small adenomatous polyp was retrieved in the ascending colon. 

The patient had an uneventful postoperative recovery, with rapid return to his usual activities. At a follow-up visit, six months later, the patient was asymptomatic, all autoantibodies tested were negative, and LAC test was negative. 

## 3. Discussion

IE remains a diagnostic challenge [2]. Up to 47 % of IE cases develop in patients without previously known heart disease [3] and in the absence of known risk factors. While early diagnosis greatly decreases complications and increases the chance of recovery, IE may present as a subacute or chronic disease with low-grade fever and nonspecific symptoms which may confuse the initial assessment and suggest a range of alternative diagnoses including rheumatologic and autoimmune diseases. In particular, *S. bovis* IE, which seems to be increasing in frequency [4], is characterized by subacute clinical features. 72% of patients with IE due to this organism do not have previously known cardiac valvular abnormalities. *S. bovis* is also associated with spondylitis (9%) and colonic tumors (53%) [4, 5]. Antiphospholipid syndrome [6] shares many clinical features with IE, including vascular thrombotic events, endocardial vegetations, and renal and cutaneous involvement. Valvular involvement, most often affecting the mitral valve, is very frequent in both primary APS [7] and in SLE-associated APS [8]. In primary APS, valvular disease was reported in 30% to 40% of patients by transthoracic echocardiography (TTE), and in 60% to 80% of patients by transesophageal echocardiography (TOE) [9]. Focal or diffuse leaflet thickening and small nodules were most commonly observed, and the presence and extent of valvular vegetations were associated with APA titers [7, 8]. 

According to the revised Sapporo criteria, the diagnosis of APS relies on the presence of clinical (vascular thrombosis or pregnancy morbidity) and laboratory criteria (presence of antiphospholipid antibodies) [1]. However, the differential diagnosis between IE and APS in individual cases may be difficult, as antiphospholipid antibodies are frequently positive during infections [10]. On the other hand, fever can be present in antiphospholipid syndrome, and cases of APS presenting as fever of unknown origin [11] or truly mimicking IE—the so-called pseudoinfective endocarditis [12]—have been described. Furthermore, APS-related valvular lesions may serve as a substrate for bacterial colonization [12]. 

The initial presentation of our patient was consistent with the diagnosis of APS, based on the presence of lupus anticoagulant, and the evidence of splenic infarcts. Leucocytosis was absent, and PCR was not raised, further suggesting a noninfectious process. The correct diagnosis was eventually made only two months later, after a high-dose corticosteroid course which might have contributed to the rapid growth of aortic vegetations, thus increasing the embolic risk of the patient [10]. However, a high degree of suspicion and strict adherence to diagnostic algorithms could have led to the correct diagnosis. In our case, the presence of fever with systemic symptoms (poor appetite, weight loss, chills), with evidence of embolism should have strengthened the suspicion of IE [2]. The modified Duke criteria are currently largely applied to the diagnosis of IE [13]: the sensitivity of the modified Duke criteria is estimated to be around 100%, with a specificity of 80% and a PPV of 100% [14]. Echocardiogram and blood cultures are the cornerstones of diagnosis and are the major criteria for the diagnosis according to Duke criteria. Both were negative in our case. However, only 1 blood culture was performed, instead of the 3 recommended by guidelines [2]. Furthermore, TTE identifies only 25% of vegetations <5 mm on native valves and 70% of vegetations >6 mm. Overall, the sensitivity of TTE on native valves is 65% while the sensitivity of TOE reaches 85 to 95%. The specificity is higher when both methods are used (90–98%) [15]. The first echocardiogram may not be sufficient for diagnostic purposes, and if clinical suspicion is high, it is recommended to perform TOE and to repeat TTE examinations at weekly intervals [2]. Furthermore, it has been reported that TTE is positive in only a minority of cases when, as in our case, few clinical criteria are present [16], and recent data suggest that TOE might be the investigation of choice in culture-negative infective endocarditis [17]. In our case, TOE was not performed for six months after the onset of symptoms. 

A final point concerns the diagnosis of APS. As it has been pointed out, the APS diagnostic criteria lack specificity and have not been adequately tested in populations other than systemic lupus erythematosus [18]. Undue reliance on APS diagnostic criteria should be avoided when infectious diseases, which can be associated with nonspecific antiphospholipid antibodies production [7], are suspected.
